# Exploring the Interplay of Telomerase Reverse Transcriptase and β-Catenin in Hepatocellular Carcinoma

**DOI:** 10.3390/cancers13164202

**Published:** 2021-08-20

**Authors:** Srishti Kotiyal, Kimberley Jane Evason

**Affiliations:** Department of Oncological Sciences, Department of Pathology, and Huntsman Cancer Institute, University of Utah, Salt Lake City, UT 84112, USA; Srishti.Kotiyal@hci.utah.edu

**Keywords:** hepatocellular carcinoma, TERT, TERT promoter, β-catenin

## Abstract

**Simple Summary:**

Liver cancer is one of the deadliest human cancers. Two of the most common molecular aberrations in liver cancer are: (1) activating mutations in the gene encoding β-catenin (*CTNNB1*); and (2) promoter mutations in telomerase reverse transcriptase (*TERT*). Here, we review recent findings regarding the interplay between TERT and β-catenin in order to better understand their role in liver cancer.

**Abstract:**

Hepatocellular carcinoma (HCC) is one of the deadliest human cancers. Activating mutations in the telomerase reverse transcriptase (*TERT)* promoter (*TERTp)* and *CTNNB1* gene encoding β-catenin are widespread in HCC (~50% and ~30%, respectively). *TERTp* mutations are predicted to increase *TERT* transcription and telomerase activity. This review focuses on exploring the role of TERT and β-catenin in HCC and the current findings regarding their interplay. TERT can have contradictory effects on tumorigenesis via both its canonical and non-canonical functions. As a critical regulator of proliferation and differentiation in progenitor and stem cells, activated β-catenin drives HCC; however, inhibiting endogenous β-catenin can also have pro-tumor effects. Clinical studies revealed a significant concordance between *TERTp* and *CTNNB1* mutations in HCC. In stem cells, TERT acts as a co-factor in β-catenin transcriptional complexes driving the expression of WNT/β-catenin target genes, and β-catenin can bind to the *TERTp* to drive its transcription. A few studies have examined potential interactions between TERT and β-catenin in HCC in vivo, and their results suggest that the coexpression of these two genes promotes hepatocarcinogenesis. Further studies are required with vertebrate models to better understand how TERT and β-catenin influence hepatocarcinogenesis.

## 1. Introduction

Hepatocellular carcinoma (HCC) forms 80% of all primary liver cancers and is the fourth leading cause of cancer-related mortality globally. Effective treatments for this disease are limited due to its molecularly heterogeneous nature [1]. Telomerase reverse transcriptase (*TERT*)*, TP53* (tumor protein p53), and *CTNNB1* encoding β-catenin are the top three most frequently mutated genes in HCC, altered in 47.1%, 29.2%, and 27.4% of cases from large-scale sequencing projects [2].

Both TERT and β-catenin play essential roles in liver development, tumorigenesis, and stemness pathways. In embryonic stem and progenitor cells, the TERT and WNT/β-catenin signaling axes crosstalk such that TERT enhances the transcription of β-catenin target genes, while β-catenin enhances the transcription of *TERT* by binding to its promoter region [3,4]. In HCC clinical specimens, activating β-catenin and *TERT* promoter (*TERTp)* mutations show significant concordance such that 74% of *CTNNB1*-mutated HCC also have *TERTp* mutations [5].

This *CTNNB1-TERT* association is special because *CTNNB1* mutations are mutually exclusive to *TP53* mutations [6] and to other perturbations in WNT pathway genes, such as *AXIN1* and *CCND1* [7]. The chromatin remodelers *ARID2* (AT rich interactive domain 2) and *ARID1A* (AT rich interactive domain 1A) are associated with mutations in *CTNNB1* (*p*-value = 0.005) and *AXIN1* (*p*-value = 0.002), respectively [6]. Potential mechanisms underlying the *ARID2-CTNNB1* and *ARID1A-AXIN1* associations in liver cancer have not been explored in depth, perhaps because these genes are less commonly altered in HCC (~10%) than *TERT* or *CTNNB1* [2].

As *TERTp* and activating β-catenin mutations are concordant in HCC and the two signaling axes directly interact in stem cells, it is crucial to define how these pathways cooperate in HCC. This review focuses on current advances in delineating the role of *TERT* and *CTNNB1* in HCC and the relationship between these two genes during carcinogenesis.

## 2. Telomeres, Telomerase, and *TERT*

Telomeres are ribonucleoprotein caps composed of short repetitive non-coding DNA sequences and associated proteins that protect the ends of eukaryotic chromosomes [8]. Telomere length varies among species and serves to mitigate two biological complications. First, together with the associated Shelterin complex, telomeres help protect DNA near chromosome ends from the DNA damage response (DDR), which is induced by double-stranded breaks in the DNA and can lead to cell cycle arrest, apoptosis, or repair. Second, telomeres provide a cushion of non-coding DNA to postpone the end replication crisis, in which genome instability results from gradual shortening of the lagging strand in semi-conservative DNA replication. Due to the presence of telomeres, chromosomes can withstand 50–90 replication cycles while losing around 50–150 nucleotides per mitosis, even in the absence of telomere maintenance mechanisms [9].

Telomerase, a multi-subunit reverse transcriptase enzyme, plays a significant role in telomere maintenance. Telomerase levels are undetectable in most somatic cells; however, this enzyme is active in certain proliferating cells, germ cells, and the majority of cancers (~90%) [10]. The telomerase holoenzyme is composed of a catalytic protein subunit encoded by *TERT*, an RNA template component (*TERC*), and auxiliary proteins. TERT consists of four main domains: the TERT N-terminal domain (TEN), the TERT RNA binding domain (TRBD), a reverse transcriptase domain (RT), and a C-terminal extension domain. The TEN domain binds to the ssDNA of telomere overhangs at the 3′ end, the TRBD domain interacts with *TERC*, and the RT and C-terminal extension domains are involved in catalyzing the addition of telomere repeats to the 3′ end and binding to the resultant DNA/RNA hybrid [8,10]. In somatic cells, *TERT* is transcriptionally repressed via epigenetic mechanisms, mainly in the *TERTp* region [9].

**Figure 1:**
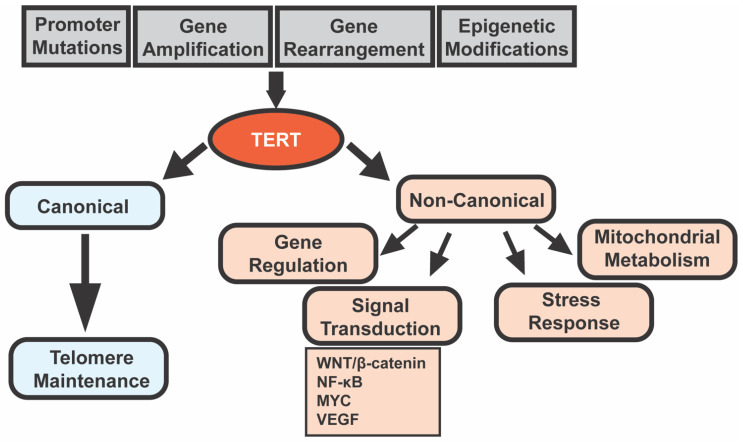
Telomerase reverse transcriptase (*TERT*) regulation and functions in cancer cells. Cancer cells may upregulate *TERT* by somatic *TERTp* mutations, *TERT* amplification and rearrangement, and/or epigenetic modifications in *TERT* and *TERTp* [8]. TERT performs both canonical (telomere lengthening) and non-canonical (gene regulation, signal transduction, stress response, and mitochondrial metabolism) functions [11,12]. Abbreviations: NF-κB, nuclear factor kappa-light-chain-enhancer of activated B cells; and VEGF, vascular endothelial growth factor.

TERT is also involved in many roles independent of its telomere lengthening activity [11]. These non-canonical functions of TERT include regulating gene expression, signal transduction, mitochondrial metabolism, and resistance to ionizing radiation [12]. Some of the most well-studied pathways regulated by TERT include the WNT/β-catenin pathway and the nuclear factor kappa-light-chain-enhancer of activated B cells (NF-κB) pathway, along with MYC, vascular endothelial growth factor (VEGF), and others [11]. TERT is found not only in the nucleus but also in the cytoplasm and mitochondria and is involved in tumorigenesis and cancer therapy resistance independent of telomere lengthening [12]. For example, the overexpression of mitochondrial *TERT* by HCC cells increases chemotherapeutic resistance (in vitro and in vivo) by increasing the mitochondrial membrane potential and inhibiting the mitochondrial apoptotic pathway [13]. The diverse roles of TERT are highlighted in Figure 1.

## 3. Telomerase and Cancer

Without a telomere maintenance mechanism, cells undergoing oncogenesis and rapid divisions gradually suffer from telomere attrition, which eventually triggers replicative senescence or apoptosis [14]. To overcome this replication barrier caused by critical telomere length, cancer cells usually increase the TERT levels and/or TERT activity via (1) *TERTp* mutations, which increase *TERT* mRNA levels by creating new ETS binding motifs [15]; (2) amplification of *TERT* and/or *TERC*; (3) *TERT* rearrangement; and/or (4) epigenetic regulation of *TERT* and its promoter [8]. As cells in high-turnover tissues have higher telomerase activity, they may be able to stably upregulate *TERT* without acquiring activating mutations like *TERTp* mutations [14]. Supporting this possibility, cancers originating from tissues with high turnover rates tend to have a lower frequency of *TERTp* mutations than those from low-turnover tissues [14].

Thus, telomerase promotes telomere maintenance, and telomere maintenance is a cancer hallmark that allows malignant cells to overcome replicative senescence. TERT also promotes cellular transformation and proliferation independent of its telomerase activity [11]. Therefore, one would predict that loss-of-function mutations in *TERT* or *TERC* would inhibit tumorigenesis, while overexpression of *TERT* would promote tumor formation. Table 1 summarizes recent findings from cancer models with manipulation of *Tert* or *Terc* in mice or zebrafish. Supporting the aforementioned prediction, *Tert* overexpression in mice leads to an increased incidence of T-cell lymphomas [16], skin papillomas [17], and mammary tumors [18], depending on the promoter used to drive its expression (Table 1). *Tert* loss of function mutants display a delayed onset of mammary tumors [19], lymphomas [20], and early HCCs [21] (Table 1).

On the other hand, *Terc* knockdown in mice results in the converse effect: enhancement of tumor formation (Table 1). *Terc^−/−^* mice treated with carbon tetrachloride (CCl_4_) or diethylnitrosamine (DEN) display increased HCC “initiation foci”—microscopic lesions with atypical cytologic or architectural features that suggest malignancy but are not sufficiently well-developed to diagnose as HCC—although HCC number and size were decreased [22]. Similarly, loss-of-function mutation of zebrafish *tert* or *terc* resulted in an earlier onset of spontaneous tumors, including intestinal adenocarcinomas, hematopoietic neoplasms, hepatocellular adenomas, and germ cell tumors [23].

Why does telomerase seem to promote tumorigenesis in some animal models yet inhibit it in others? One possible explanation is that long-term inhibition of *Tert* or *Terc*, as in these genetic animal models, results in compensation by other telomere maintenance programs, such as alternative lengthening of telomeres (ALT). ALT is a telomerase-independent mechanism for telomere maintenance. ALT-mediated telomere synthesis occurs via homologous recombination between non-allelic telomeres, sister chromatids at telomeric sites, and/or linear or circular extrachromosomal telomeric repeats [24]. This compensatory mechanism might not be relevant to human hepatocarcinogenesis, during which *TERT* could potentially be inhibited more acutely using drugs. Inducible and/or conditional animal models in which *Tert* and/or *Terc* can be turned on at different time points during tumorigenesis would be helpful to dissect the importance of acute versus chronic telomerase inhibition.

Another possible reason why telomerase might inhibit tumorigenesis in some animal models while promoting it in others is related to the balance between canonical and non-canonical effects of *Tert.* Laboratory mouse models have long telomeres (25–40 kb vs. 10–15 kb in humans) that are likely sufficient to permit malignant transformation even in the absence of telomerase enzyme [12]. Therefore, *Tert* overexpression and mutant phenotypes in mouse tumor models may be at least partially independent of *Tert* telomere lengthening activity [16,17,18]. As discussed in the section above, non-canonical functions of TERT tend to be tumorigenic and anti-apoptotic. Thus, *Tert* deficiency in mice may delay tumorigenesis due to the resultant reduction in its non-canonical activity [19,21].

Zebrafish have telomere lengths similar to humans (~5–15 kb) [25] and, like humans, exhibit drastic shortening of telomeres with age [23]; therefore, the manipulation of *tert* levels is more likely to impact telomere length than in mice. Thus, the canonical effects of *tert* may be relatively more important to its role in zebrafish carcinogenesis. Zebrafish *tert* deficiency promotes the initiation of tumorigenesis through shortened telomeres that induce DNA damage responses, inflammation, and genetic instability [23]. A similar phenomenon occurs in aged late-generation *Terc^−/−^* mice, wherein shortened telomeres are associated with ulcerated skin lesions, defective wound healing, and genomic instability [26].

These findings highlight that *TERT* influences tumorigenesis in both canonical and non-canonical ways. Whether the net effects of *TERT* are pro- or anti-tumor depends on the stage of disease, tissue type, telomere biology in different animal models, when during tumor formation *TERT* is manipulated, and other factors. Additional studies of vertebrate HCC models, including rigorous examination of telomere length during carcinogenesis, could help define the importance of TERT’s telomerase activity to HCC pathogenesis.

**Table 1:** TERT cancer models in mice and zebrafish.

Gene (Animal)	Site Specificity	Expression *	Result **	Ref.
*Tert* (mouse)	Thymocytes and peripheral T cells	+	↑ T-Cell Lymphomas	[16]
*Tert* (mouse)	Basal keratinocytes	+	↑ skin papillomas (DMBA + TPA induction)	[17]
*Tert* (mouse)	Whole body	+	↑ mammary tumors in aged females	[18]
*tert* and *terc* (zebrafish)	Neural progenitor cells	+	↓ aggressiveness of RAS-mediated brain tumors	[27]
*Tert* (mouse)	Whole body	−	Delayed onset of lymphomas in EμMYC mice	[20]
*Tert* (mouse)	Whole body	−	Delayed onset of mammary tumors in PyMT mice	[19]
*Tert* (mouse)	Whole body	−	↓ HCC “initiation foci” (CCl_4_ induction)	[21]
*Terc* (mouse)	Whole body	−	↑ HCC “initiation foci” but ↓ HCC progression (uPA, CCl_4_ or DEN induction)	[22]
*Terc* (mouse)	Whole body	−	↑ tumors (lymphomas, teratocarcinomas, HCC, squamous cell carcinoma)	[26]
*Terc* (mouse)	Whole body	−	↓ skin papillomas (DMBA + TPA induction)	[28]
*Terc* (mouse)	Whole body	−	↑ epithelial cancers in TP53^−/−^ mice	[29]
*Terc* (mouse)	Whole body	−	↑ adenoma initiation but ↓ progression in *Apc^Min^* mice	[30]
*tert^hu3430^* (zebrafish)	Whole body	−	Earlier onset of tumors (germ cell tumors, hematopoietic neoplasms, HCA, etc.)	[23]
*tert^hu3430^* (zebrafish)	Whole body	−	↑ tumor incidence and aggressiveness of melanoma model ***	[31]

*: +, gene overexpression; −, gene knockout or knockdown. **: ↑, increased; ↓, decreased. *** *mitfa*: HRAS (gives rise to melanomas) blastula cells transplanted into *tert^−/−^* casper embryos. Abbreviations: DMBA, 7,12-dimethylbenz[*a*]anthracene; TPA, 12-*o*-tetradecanoylphorbol 13-acetate; PyMT, polyomavirus middle T oncogene; CCl_4_, carbon tetrachloride; uPA, urokinase plasminogen activator; DEN, diethylnitrosamine; HCA, hepatocellular adenoma; *Apc*, adenomatous polyposis coli; *Apc^Min^*, multiple intestinal neoplasia (mutant) allele of *Apc* gene; and *tert^hu3430^*, *tert* mutant line (allele hu3430) with a non-sense mutation resulting in a premature stop codon in exon 2 of *tert* gene.

## 4. *TERT* Promoter Mutations in HCC

HCC is an extremely heterogeneous disease with its prevalence, etiology, and epidemiology showing marked variance across cohorts separated by geography, ancestry, and sex. Eighty percent of HCCs arise in a cirrhotic background caused by various insults, including hepatitis C virus (HCV) and hepatitis B virus (HBV) infections, alcohol intake, non-alcoholic steatohepatitis (NASH), and aflatoxin exposure [32]. Less commonly, HCC occurs via malignant transformation in the non-cirrhotic liver or hepatocellular adenoma (HCA) [32]. The variable etiological and epidemiological causes contribute to the molecularly heterogeneous nature of HCC [32].

*TERT* re-expression is observed in approximately 90% of HCC cases [33]. Genetic alterations in *TERT* can be due to *TERTp* mutations (54% of cases), *TERT* focal amplification (6.7%), or HBV genome integration into the *TERT* promoter (22% of HBV-related HCC) [7]. *TERTp* mutations are mutually exclusive with *TERT* focal amplifications and HBV genome integration in HBV positive cases [7].

*TERTp* mutations in HCC typically involve one of two mutational hotspots of cytosine to thymine transitions, 1,295,228 C > T (termed C228T) and 1,295,250 C > T (C250T) at −124 bp and −146 bp, respectively [34]. Each of these mutually exclusive mutations creates de novo binding sites for ETS transcription factors within the *TERT* promoter. These mutations are predicted to increase *TERT* expression levels, enzymatic activity, and telomere length; they are also associated with decreased survival in cancer patients [35,36,37,38].

For example, *TERTp* mutations significantly increased TERT mRNA levels in human malignant pleural mesothelioma samples (only C228T mutation detected; *p*-value = 0.02) [39] and were significantly correlated with thyroid cancer mortality (83% C228T, 17% C250T; *p*-value < 0.001) [40]. These mutations are linked to increased TERT expression (*p*-value < 0.0001 and *p*-value = 0.007) in HCC patients as well [5,41].

In accordance with HCC heterogeneity, the prevalence of *TERTp* mutations also varies based on etiological/epidemiological factors. We performed a PubMed search using the search terms “TERTp”, “mutations”, and “hepatocellular carcinoma”. We reviewed all studies using archived tumor genomic DNA or formalin fixed tumor tissue samples with matching non-tumor controls. Table 2 summarizes these studies, delineating the prevalence of *TERTp* mutations across diverse geographies and etiologies. This table focuses on the two common hotspot mutations mentioned in the above paragraph and excludes other rare *TERTp* mutations, if detected. Overall, *TERTp* mutations are enriched in HCV-related HCC compared to HBV or non-viral HCC cases [7,35,42,43,44,45,46] and may be associated with the male sex [5,35,44]. C228T mutations are the dominant *TERTp* mutation regardless of etiology (Table 2).

An analysis of 1061 HCC genomes from four different geographical locations showed a significant link between *TERTp* mutations and more aggressive tumors (*p*-value = 1.02 × 10^−5^), relapse (*p*-value = 2.33 × 10^−12^), and decreased overall survival (*p*-value = 2.81 × 10^−5^) [47].

**Table 2:** Landscape of *TERTp* mutations in HCC.

Region	Etiology (N)	C228T Incidence % (N)	C250T Incidence % (N)	Association *	Ref.
(1) Asia (2) Africa (3) Europe	52.3% HBV, rest unknown (regions 1–3) (44)	(1) 16% (3) (2) 33% (5) (3) 20% (2)	(1) 5% (1) (2) 20% (3) (3) 10% (1)	↑ frequency in Africans vs. non-Africans (*p* = 0.056) and in HBV− vs. HBV+ (*p* = 0.295) (regions 1–3)	[48]
Southern Italy	(67)	41.8% (28)	0% (0)	↑ *TERT* expression in mutated tumors vs. control tissue (*p* < 0.0001)	[41]
Southern Italy	7.9% HBV+, 86.6% HCV+ (127)	48.8% (62)	1.6% (2)	↑ frequency in HCV+ vs. HBV+ (*p* < 0.001)	[43]
Germany	(78)	47.4% (37)			[49]
France, Italy and Spain	14% HBV+, 26% HCV+ (243)	54.3% (132)	2% (5)		[6]
France	22% HBV+, 26% HCV+ (305)	55% (168)	3.6% (11)	↑ frequency in males vs. females (*p* = 0.001) and in HBV− vs. HBV+ (*p* < 0.0001); ↑ *TERT* expression in mutated HCC vs. normal liver, cirrhosis, and HCA (*p* = 0.0007)	[5]
USA	24.6% HBV+, 26% HCV+ (61)	42.6% (26)	1.6% (1)	no correlation with etiology, sex, age, or ethnicity	[50]
(1) USA (2) Japan	(1) 14.6% HBV+, 57% HCV+ (89) (2) 28.6% HBV+, 37% HCV+ (374)	(1) 34.8% (31) (2) 55.6% (208)	(1) 2.2% (2) (2) 2.4% (9)	↑ frequency in HCV+ vs. HCV− (*p* = 0.0016)	[7,42]
China	94% HBV+ (276)	30.5% (84)	0.36% (1)	↑ frequency in older vs. younger age (*p* = 0.04); no correlation with sex or etiology	[51]
China	(35)	25.7% (9)	5.7% (2)		[52]
China	83% HBV+ (190)	26.3% (50)	3.7% (7)	no correlation with age, sex, etiology, or tumor status	[53]
Japan	(11)	81.8% (9)			[54]
Japan	23% HBV+, 61.6% HCV+ (125)	66.4% (83)	1.6% (2)	↑ frequency in HCV+ vs. HCV− (*p* = 0.0007) and in viral vs. non-viral (*p* = 0.0282)	[45]
South Korea	36% HBV+, 3% HCV+ (160)	20% (32)	8.75% (14)	↑ frequency in males vs. females (*p* = 0.027) and in HCV+ vs. HCV− (*p* = 0.285); no association with telomere length or HCC prognosis	[35]
South Korea	74% HBV+, 5.7% HCV+ (105)	37% (39)	1.9% (2)	↑ frequency in HCV+ vs. HCV− (*p* = 0.001)	[46]
Taiwan	63% HBV+, 40.2% HCV+ (195)	27.7% (54)	1.5% (3)	↑ frequency in HCV+ vs. HCV− (*p* = 0.0048), older vs. younger age (*p* = 0.0122), and HBV− vs. HBV+ (*p* = 0.0007)	[34]
USA, Canada, South Korea, Vietnam, and Russia	22.4% HBV+, 17.8% HCV+ (196)	40.8% (80)	3.6% (7)	↑ frequency in older vs. younger age (*p* = 0.0006), males vs. females (*p* = 0.006), HCV+ vs. HCV− (*p* = 0.04), and HBV− vs. HBV+ (*p* = 0.02); no association with *TERT* expression	[44]

*: ↑, increased or higher.

Apart from the two hotspot mutations (C228T and C250T), the *TERT* promoter SNP rs2853669 is also common in several cancers and is predicted to decrease *TERT* expression [55]. However, little is known of its role in HCC. Ko et al. analyzed Korean HCC samples and observed that the combination of rs2853669 with *TERTp* mutation decreased patient survival and increased cancer recurrence risk. The SNP alone had no effect. This combination led to increased *TERT* expression and telomere length compared to rs2853669 only [56].

A study from Southern Italy analyzed 84 HCC cases and did not find a correlation between *TERTp* mutations and survival with or without the SNP [43]. However, the frequency of SNP genotypes was not in Hardy–Weinberg equilibrium in cases with *TERTp* mutations, indicating the presence of selective pressure for the combination of *TERTp* mutation and the rs2853669 SNP [43]. Other studies have noted a high prevalence of this SNP in HCC patients with no link to increased TERT expression or *TERTp* mutational frequency [41,57]. To better understand the prognostic value of this SNP’s association with *TERTp* mutations, further clinical studies with larger and diverse (in terms of etiology, sex, driver mutations, etc.) cohorts are needed as well as more mechanistic studies.

## 5. WNT/β-Catenin Signaling in Normal and Cancer Cells

**Figure 2:**
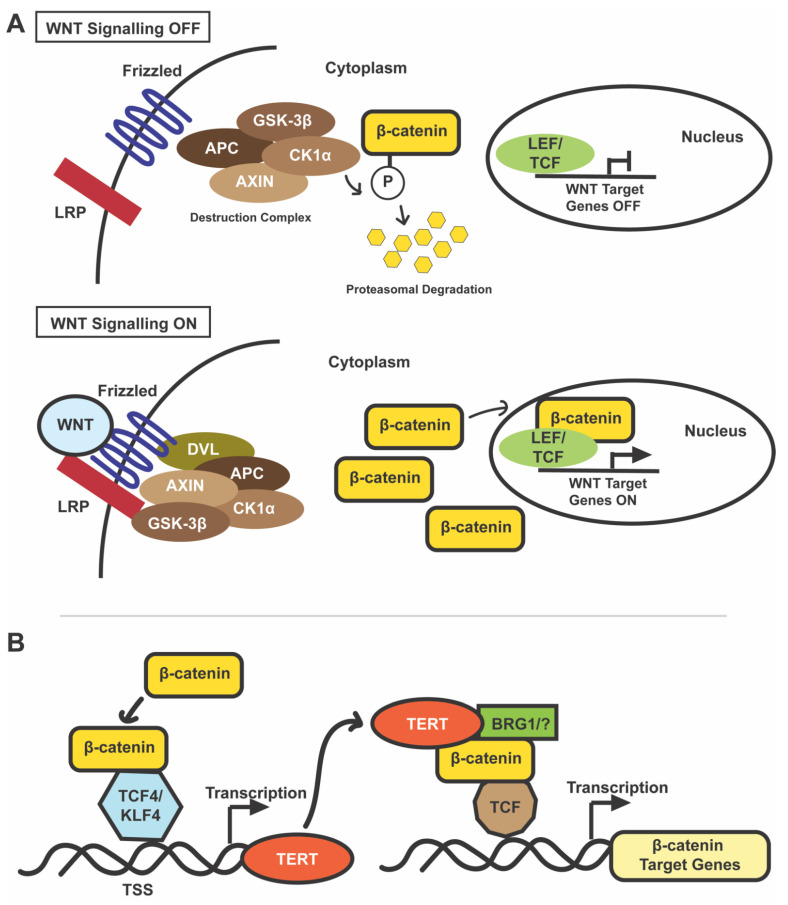
(**A**) Canonical WNT/β-catenin pathway. When WNT signaling is OFF (top), cytoplasmic β-catenin is phosphorylated by a destruction complex comprising of APC, AXIN, GSK-3β, and CK1α [58,59]. This phosphorylation marks it for proteasomal degradation, and consequently it is unavailable to bind to LEF/TCF elements in the nucleus to drive transcription of target genes [58,59]. When WNT signaling is ON, WNT ligands bind to LRP and Frizzled receptors leading to the destabilization of the destruction complex by a mechanism that may involve DVL protein [58,59]. The degradation complex is, thus, unable to phosphorylate and degrade cytoplasmic β-catenin [58,59]. β-catenin accumulates in the cytoplasm and then enters the nucleus to bind to TCF/LEF elements and drive transcription of WNT target genes [58,59]. (**B**) Direct interplay of TERT and β-catenin signaling. β-catenin enhances transcription of TERT by binding to its promoter region [4]. In mouse ES cells, this β-catenin-TERT interaction is facilitated by Klf4 [4]; another study showed that β-catenin induced an increase in TERT mRNA and telomerase activity is dependent on TCF4 binding to *TERTp* [62]. TERT modulates WNT signaling by interacting with BRG1 (a chromatin-remodeling protein; “?” indicates uncertainty as some groups did not observe this association), binding to the promoter of WNT responsive genes, and acting as a co-factor [3]. This transcriptional upregulation of WNT target genes by TERT is independent of its catalytic activity [3]. Abbreviations: APC, adenomatous polyposis coli; CK1α, casein kinase 1α; GSK-3β, glycogen synthase kinase 3β; and DVL, disheveled.

The WNT/β-catenin signaling pathway, depicted in Figure 2A, is a critical regulator of the proliferation and differentiation of stem and progenitor cells [58]. In the absence of WNT ligands, a destruction complex comprising adenomatous polyposis coli (APC), AXIN, glycogen synthase kinase 3β (GSK-3β), and casein kinase 1α (CK1α) phosphorylates cytoplasmic β-catenin at its N-terminal serine/threonine residues. This phosphorylation triggers its ubiquitination followed by proteasomal degradation [58,59]. As cytoplasmic β-catenin gets degraded, it cannot accumulate and translocate into the nucleus to drive the expression of WNT target genes. 

In the presence of WNT ligands, binding of the receptor complex destabilizes the destruction complex and leads to the stabilization and cytoplasmic accumulation of β-catenin [58,59]. β-catenin then undergoes nuclear translocation where it binds to T cell factor/lymphoid enhancer factor (TCF/LEF) transcription factors and replaces the transcriptional repressor Groucho. This complex formation drives the expression of WNT target genes, such as *C-MYC, EPCAM, CYCLIN D1*, and *CD44*, which promote cell cycle progression, proliferation, metabolism, cell survival, immune tolerance, and angiogenesis [58,59].

As a central regulator of the WNT cascade, β-catenin levels can be increased in cancer via stabilizing mutations, which increase the nuclear accumulation of β-catenin and upregulate WNT/β-catenin signaling [60,61]. Exon 3 is a mutational hotspot for such stabilizing missense mutations that target the serine/threonine sites that render β-catenin resistant to GSK-3β mediated phosphorylation and, thereby, to subsequent proteasomal degradation. Some of the most common mutational sites in this hotspot are S33, S37, S45, T41, D32, and G34 [60]. Activating mutations in *CTNNB1* have been reported in several cancers, including colorectal cancer, HCC, endometrial cancer, melanoma, ovarian cancer, and thyroid cancer [61].

## 6. WNT/β-Catenin in HCC

The WNT/β-catenin pathway is integral for liver development and homeostasis [59]. Its dysregulation is linked to various hepatic diseases, including HCC. Aberrant WNT/β-catenin signaling has been detected in up to 66% of HCC cases (mutually exclusive mutations in *CTNNB1*, *AXIN1*, and *APC*, and *CCND1*-*FGF19* amplification) [7]. Deletions or missense mutations in the *CTNNB1* gene are the most frequent WNT/β-catenin dysregulating event in HCC [7]. In terms of etiology, *CTNNB1* mutations are strikingly more prevalent in HCV-related HCCs (~26.7%) than in HBV-related HCC (11.6%) or non-viral HCC (21.2%) (*p*-value < 0.0001) [63]. These percentages may vary between studies depending on the cohorts, but *CTNNB1* mutations are unanimously higher in HCV-related HCCs than HBV- or non-viral-related HCCs [64].

The results are conflicting concerning the prognostic value of *CTNNB1* mutations. Khalaf et al. summarized clinical studies linked to β-catenin and HCC and their outcomes [64]. Certain studies indicate that the presence of *CTNNB1* mutations is associated with decreased aggressiveness, invasiveness, serum α-fetoprotein levels, and more differentiated HCC [64,65,66,67,68,69], while others linked it to poorer prognosis (small vessel invasion and tumor capsule invasion) or no effect on survival [64,70].

Gain and loss of function studies in vertebrate models have helped to elucidate the role of *CTNNB1* mutations in HCC initiation, progression, and maintenance. Table 3 summarizes how overexpressing (+) or knocking down (−) wild-type or mutant *CTNNB1* in hepatocytes affects HCC. Studies led by Satdarshan Monga, Makoto Taketo, and Christine Perret have indicated that the expression of wild-type or activated forms of β-catenin in mouse hepatocytes under control of the *Alb* (albumin) or *Fabp* (fatty acid-binding protein) promoter is not enough to drive HCC [71,72,73,74] (Table 3). Similarly, targeting mouse hepatocytes with activated β-catenin by hydrodynamic tail vein injection (HDT) is not sufficient to induce HCC [75,76,77] (Table 3). 

On the other hand, activated β-catenin can collaborate with other oncogenes or chemical insults to drive HCC in mice [72,75,76,77,78] (Table 3). In addition, expression of activated β-catenin in mouse liver progenitor cells or zebrafish hepatocytes is sufficient to induce HCC [79,80,81]. Together these studies indicate that activating β-catenin mutations promote HCC, although additional genetic or epigenetic alterations may be required for HCC initiation or progression in some contexts, particularly if activated β-catenin is turned on after early development.

While activated β-catenin drives HCC, endogenous β-catenin plays a restraining role in hepatocarcinogenesis in at least some contexts. Knockout of endogenous β-catenin is pro-tumorigenic in mouse DEN-induced hepatocarcinogenesis, an effect that is abrogated by treatment with the antioxidant *N*-acetyl-L-cysteine (NAC) [82]. Knockout of endogenous β-catenin also promotes mouse HCC induced by hydrodynamic tail vein injection (HDT) with activated β-catenin and MET [83]. Gene expression analysis of HCC lacking endogenous β-catenin showed an enrichment of inflammatory and DNA damage response genes [83]. 

Together, these studies suggest that inhibiting endogenous β-catenin may promote HCC by creating a pro-inflammatory, pro-oxidant microenvironment. As therapeutic targeting of β-catenin becomes more widespread the clinic, it will be critical to keep the anti-oncogenic effects of endogenous β-catenin in mind, as they may influence recurrence after targeting the primary tumor.

**Table 3:** HCC animal models with *CTNNB1* manipulation.

Animal	Method *	Expression **	*CTNNB1* Status ***	Results	Ref.
Mice	*Alb* promoter	+	Wildtype	Transgenic mice develop hepatomegaly but no tumors	[71]
	*Alb* promoter	+	Ser45 mutated	Transgenic mice develop hepatomegaly (only in younger mice) but no tumors; Increased HCC (DEN induction)	[72]
	*Fabp: Cre*	+	Exon 3 deleted	Transgenic mice develop hepatomegaly but no tumors	[73]
	*CaBP9K* promoter	+	N131 deleted	Transgenic mice develop hepatomegaly but no tumors	[74]
	*Cited1: CreER*	+	Exon 3 deleted	Transgenic mice develop HCCs, hepatoblastomas, and lung metastases	[79]
	AdCMV-*Cre*	+	Exon 3 deleted (and *H-RAS*)	Double transgenic mice develop HCC, but no HCC develops with expression of mutated *CTNNB1* alone	[78]
	SB-HDT	+	S45 or S33 mutated (and *MET*)	Mice expressing *MET* and either form of mutated *CTNNB1*, but not mutated *CTNNB1* alone, develop HCC	[75]
	SB-HDT	+	N90 deleted (and activated *YAP*)	Mice expressing activated *YAP* and mutated *CTNNB1*, but not mutated *CTNNB1* alone, develop HCC	[76]
	SB-HDT	+	S45 or S33 mutated (and *K-RAS*)	Mice expressing *K-RAS* and either form of mutated *CTNNB1*, but not mutated *CTNNB1* alone, develop HCC	[77]
	siRNA	−	S45 mutated (HDT induction)	Decreased HCC (*K-RAS* plus S45-mutated *CTNNB1* HDT induction)	[77]
	*Alb:Cre*	−	Wildtype (endogenous)	Increased HCC (DEN induction)	[82]
	*Alb:Cre*	−	Wildtype (endogenous)	Increased HCC (*MET* and N90-deleted *CTNNB1* induction)	[83]
Zebrafish	*fabp10a* promoter	+	S33A, S37A, T41A, and S45A mutated	Transgenic zebrafish develop HCC as adults (78% by 6 months)	[81]
	*fabp: CreERT2*	+		Transgenic zebrafish develop HCC as adults (13% by 6 months)	[80]

*: Method indicates promoter or method used to express or target *CTNNB1*. **: +, *CTNNB1* overexpression or expression of activated form of *CTNNB1*; -, *CTNNB1* knockout or knockdown. ***: For gain-of-function (+) studies, *CTNNB1* status indicates the wildtype or activated form of the gene that is expressed in each study. Other genes targeted in the model are shown in parentheses. For loss-of-function (−) studies, the form of the targeted *CTNNB1* allele is shown, with its origin indicated in parentheses. Abbreviations: *Alb*, albumin; *Fabp*, fatty acid-binding protein; *Fabp: Cre, Fabp* promoter driving expression of Cre recombinase; *CaBP9K*, calbindin-D9K; *Cited1: CreER*, Cited1 driving expression of tamoxifen-inducible Cre (CreER); AdCMV-*Cre*, recombinant adenovirus with cytomegalovirus promoter driving Cre recombinase; SB-HDT, sleeping beauty transposase-mediated hydrodynamic transfection; *Alb*: Cre, albumin promoter driving Cre recombinase; and DEN, diethylnitrosamine.

In patient HCC samples with activating mutations in β-catenin, immunostaining for cytoplasmic and nuclear β-catenin—indicative of active WNT/β-catenin signaling—is typically patchy or focal, confined to a small percentage of tumor cells [70]. This observation seems to result from actual differences in WNT/β-catenin signaling activity among tumor cells rather than from a lack of antibody sensitivity. Our lab has shown that WNT reporter activity and expression levels of WNT/β-catenin target genes are highly variable from cell to cell in zebrafish β-catenin-driven HCC [80,81].

Furthermore, expression of activated β-catenin in even a small subset of larval hepatocytes is sufficient to drive HCC in zebrafish [80]. Similar results were reported by Mokapatti et al., who found that expression of activated β-catenin in hepatic progenitor cells (4% of fetal liver cells) initiated HCC in mice [79]. These results highlight the importance of β-catenin heterogeneity in the initiation and progression of HCC.

## 7. Interactions between TERT and β-Catenin in Cultured Cells and Animal Models

Manipulations in *TERT* or WNT/β-catenin signaling result in strikingly similar phenotypes in mouse skin, suggesting these two pathways may cooperate in this system. Sarin et al. investigated the role of *TERT* in multipotent stem cells/progenitor cells by conditional *TERT* expression in mouse skin [84]. The hair follicle region consists of multipotent stem cells and progenitor cells, which cycle from telogen phase (resting phase) to anagen phase (active phase) [84]. *TERT* modulates this transition by increasing the proliferation of the stem cell population [84].

This anagen-promoting effect is independent of the telomerase activity of TERT, as these effects are retained even in the absence of *TERC* [84] and mirror the phenotype of β-catenin overexpression in hair follicles [85,86,87,88]. These results highlight the similarities in function between non-canonical TERT activity and the WNT/β-catenin pathway in stem cells. Choi et al. further underlined that TERT regulates stem cell activity independently of its telomerase activity by overexpressing a catalytically inactive form of TERT (TERT^ci^) [89]. TERT^ci^ had the same effect as TERT in promoting anagen in hair follicles and keratinocyte proliferation in the skin. They found strong associations between TERT and WNT/β-catenin pathways through genome-wide analysis of transcriptional changes wherein TERT specifically regulated WNT target genes with TCF/LEF elements [89].

Studies predominantly performed in mouse stem cells delineate a feedback loop wherein Wnt/β-catenin signaling and TERT upregulate each other (Figure 2B). A key paper in this field by Park et al. illustrated that TERT regulates WNT/β-catenin signaling by acting as a co-factor in the β-catenin transcription complex [3]. They found that, in cultured mouse ES cells, endogenous TERT associated with the bromodomain of BRG1 to regulate transcription of β-catenin target genes [3]. TERT overexpression hyperactivated WNT/β-catenin signaling independent of TERT catalytic activity [3].

In mouse stomach cells, endogenous TERT occupied TCF binding elements together with β-catenin, and TERT^ci^ overexpression increased promoter activity and/or expression of β-catenin target genes, such as *AXIN2* and *CD44* [3]. TERT knockout in mouse ES cells decreased both the basal and WNT3A-induced expression of WNT/β-catenin target gene *AXIN2* [3]. Corroborating Park et al., Hrdlickova et al. showed that the overexpression of TERT/TERT^ci^ splice variants increased proliferation and activated WNT signaling in TERT-deficient and TERT-expressing human cell lines [90].

Hoffmeyer et al. described the role of WNT/β-catenin signaling in regulating *TERT* in stem cells and cancer cells [4]. Using ES cells, they showed that β-catenin knockdown resulted in reduced *TERT* (but not *TERC*) mRNA levels as well as decreased telomerase activity while its overexpression increased *TERT* mRNA, protein, and telomerase activity [4]. Telomere length increased upon β-catenin overexpression and decreased in β-catenin-deficient cells relative to wildtype. In terms of this upregulation mechanism, β-catenin binds at the transcriptional start site (TSS) of *TERT*, possibly by forming a complex that requires Klf4 [4]. These results indicate that β-catenin promotes *TERT* transcription in ES cells [4]. Similarly, β-catenin was found at the TSS of *TERT* in adult mouse stem cells, primary mouse neurospheres, and human cancer cell lines, correlating with *TERT* expression [4].

Similar results were observed by Zhang et al. in 293T (human embryonic kidney), HCT116 (human colon cancer), and MCF7 (human breast cancer) cell lines wherein WNT/β-catenin upregulates *TERT* expression levels and telomerase activity [62]. Mechanistically, they found that TCF4 (and not TCF1/3 or LEF1) was involved as a binding partner of β-catenin, responsible for increasing *TERT* promoter activity [62]. The WNT/β-catenin target MYC also binds at the TSS of *TERT* to increase its expression [91,92]. Another study using human colorectal Caco2 and SW620 cells showed that β-catenin directly regulated *TERT* via TCF4 binding, and this effect was independent of MYC [93].

Some groups have failed to discern interactions between *TERT* and WNT/β-catenin during transcriptional regulation. Listerman et al. overexpressed *TERT* in breast cancer cell lines with varying endogenous levels of *TERT* expression and saw WNT/β-catenin signaling activation only in those cell lines that had low endogenous telomerase activity [94]. They did not observe an interaction between FLAG-TERT and β-catenin or BRG1 and proposed that the results seen by Park et al. were due to their anti-FLAG antibody binding to a molecule other than β-catenin but with similar electrophoretic mobility [94].

Additionally, Strong et al. did not find abnormalities in WNT/β-catenin-signaling-related developmental processes in *Tert^−/−^* mice [95]. Their TOPFLASH assay did not reveal any difference in WNT activation levels in *Tert^−/−^* mice vs. wildtype [95]. Similarly, another study in *Tert^−/−^* and *Terc^−/−^* G1 mouse embryonic fibroblasts reported no transcriptional profile changes, including WNT/β-catenin target genes, compared to wildtype [96]. Possible reasons for these contradictions could be germline compensation in WNT/β-catenin signaling in these mutants [11], different mouse backgrounds, different epitope tags or antibodies used, and different assay sensitivities.

WNT and TERT are also linked by their relationship to telomere capping [97,98]. Late generation *Terc^−/−^* mice show telomere shortening leading to uncapping. These capping defects can downregulate WNT/β-catenin signaling in high turnover cells, and WNT agonists can rescue the effects [98]. This rescue is not mediated by telomere lengthening but by increased expression of Shelterin complex proteins (*Trf2* and *Pot1a)* [98]. This group proposed that the phenotype observed by Park et al. may be due to the presence of a shortened telomere in the *Tert^+/−^* parent that led to the downregulation of WNT/β-catenin signaling at some critical phase of development of the G1 *Tert^−/−^* offspring [98]. These differences highlight that the impact of *TERT* overexpression or deletion on WNT/β-catenin signaling is not a universal effect and may depend on telomere length, developmental stage, cell type, or other factors.

## 8. TERT and β-Catenin in Human Cancer

Associations between *TERT* and β-catenin have been reported in diverse cancer types, including gastrointestinal cancers, medulloblastoma, breast cancer, and osteosarcoma. The expression of *CTNNB1*, its target genes, and *TERT* have been reported at the invasive edges of colorectal cancer [93]. Jaitner et al. analyzed 24 human colorectal cancer samples with invasive fronts and found that *TERT* expression was higher in tumor cells with nuclear β-catenin, while tumor cells without nuclear β-catenin did not express TERT [93]. Similarly, significant protein–protein interactions between β-catenin and TERT were detected in 104 esophageal cancer patient samples [99].

In gastric cancer cell lines, *TERT* overexpression enhances WNT/β-catenin signaling and increases the expression of WNT/β-catenin target genes, such as *c-MYC* and *Cyclin D1* [100]. This *TERT*-induced *c-MYC* upregulation increases the expression of heparanase, a metastasis-promoting protein, leading to increased aggressiveness and poor prognosis [100]. *TERT* mutations are recurrent in the WNT subgroup of medulloblastoma although they do not impact prognosis [101]. In breast cancer, *TERT* can upregulate WNT signaling but only in a cell line dependent manner: out of four cell lines tested, only one showed WNT reporter activity in response to *TERT* overexpression [94]. *TERT*-induced miR-500a upregulation increased tumor aggressiveness in osteosarcoma cell lines, and WNT/β-catenin is predicted to be one of the affected downstream signaling pathways [102].

## 9. TERT and β-Catenin in HCC Patients

Several small- and large-scale genome sequencing efforts in HCC samples with diverse etiologies, summarized in Table 4, provide clues regarding the association between the two genes. A seminal study by Nault et al. illustrated the concordance between TERT and β-catenin while also indicating their interplay in HCC initiation and progression [5]. They screened 305 HCC cases from two French hospitals and found that 59% of cases had *TERTp* mutations, significantly correlated with *CTNNB1* mutations (*p*-value < 0.0001, *χ*^2^-test) [5]. The same group further confirmed this association in a study with 720 patients (*p*-value = 0.0000001) [103].

**Figure 3:**
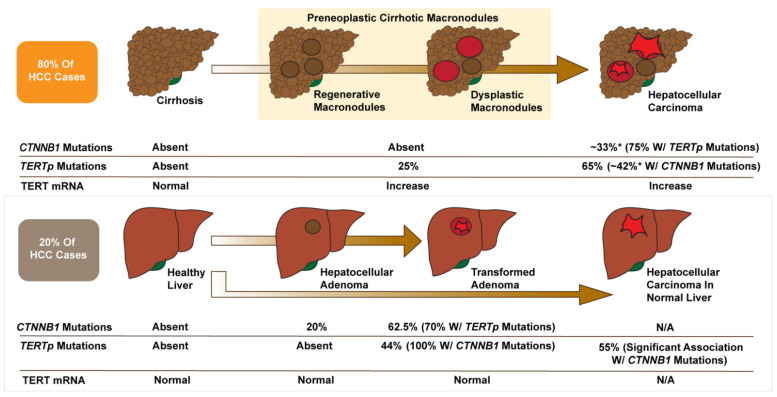
Multi-step hepatocarcinogenesis: Interplay of *TERTp* and *CTNNB1* mutations. Adapted from Nault et al. and Pinyol et al. [5,105]. *TERTp* mutations (found in the majority of HCC cases) are the earliest somatic alteration found in preneoplastic cirrhotic lesions and are associated with increased *TERT* transcription. Activating *CTNNB1* mutations and *TERTp* mutations show high concordance in HCC, and *CTNNB1* mutations are enriched in later stages of malignancy, indicating their role as cancer drivers. In HCA, *TERTp* mutations might flip the switch from benign β-catenin-linked tumorigenesis to malignant HCC. *: Mutation incidence in total HCC samples irrespective of cirrhotic or non-cirrhotic background. N/A: Not applicable as not reported separately in large-scale sequencing studies.

For HCC arising in cirrhotic livers, *TERTp* mutations seemed to be the first genetic alteration leading to malignancy, being found in 25% of preneoplastic lesions compared to 65% of malignant tumors (42% of these with concurrent β-catenin mutations) (Figure 3) [5]. *TERTp* mutations were absent in cirrhotic livers without preneoplastic lesions but enriched as the cirrhotic tissue progressed into dysplastic nodules and HCC, correlating with increased *TERT* expression (Figure 3) [5]. These results were corroborated in another study by Torrecilla et al. that identified *TERT* as the gatekeeper mutation and *CTNNB1* as a driver mutation in cirrhotic hepatocarcinogenesis in patient samples from the USA, Italy, and Spain [104].

For HCC arising from HCA, *CTNNB1* mutations appear to be the initiating event, being found in 20% of HCA without malignant transformation (0% with *TERT* mutations) (Figure 3) [105]. β-catenin activated HCAs, which are characterized by activating *CTNNB1* mutations, nuclear β-catenin staining, and strong diffuse staining for the WNT/β-catenin target gene *GLUL*, are significantly more likely to transform to HCC than other HCA subtypes [106,107]. In transformed adenomas, 44% have *TERTp* mutations, all associated with β-catenin mutations (Figure 3) [5,105]. These studies suggest a gatekeeping role of *TERT* in HCA: β-catenin mutations are an early event driving proliferation and a significant risk factor for malignancy; however, *TERTp* mutations are critical for malignant transformation [108,109,110].

A study done on a European cohort with a high prevalence of HCV-related HCC also showed the coexistence of *TERTp* and *CTNNB1* mutations, as 57.6% of *CTNNB1*-mutated HCC had *TERTp* mutations (*p*-value = 0.4192) [43]. Another study with Asian (Japanese) and American (mixed ancestry) populations observed significant concordance between *TERTp* mutations and WNT pathway alterations (*CTNNB1*, *AXIN1*, or *APC*) in HCV and non-virus related HCCs (*p*-value < 0.001) [7].

In HCC cases arising in patients with non-alcoholic fatty liver disease (NAFLD), *TERTp* mutations are very high and more frequent than in HCC associated with HBV or even HCV [42,54]. In a Japanese study, all NAFLD-HCC with *CTNNB1* mutations were mutated for *TERTp*; however, the association was statistically insignificant (*p*-value = 0.4545), likely due to the small sample size of 11 [54].

The aforementioned studies all demonstrated a high concordance between *TERTp* and β-catenin mutations; however, none of them found evidence to support the direct regulation of one gene by the other [5,7,43,54,103]. In HCC with *TERTp* mutations and not *CTNNB1* mutations, β-catenin target genes (*GLUL* and *LGR5)* were not upregulated compared to HCC without mutations in *TERT* or β-catenin [5]. In HCA with *CTNNB1* mutations, *TERT* transcription was not increased [5].

Not all clinical studies have identified an association between *TERTp* and β-catenin mutations. Studies done on Taiwanese, Korean, and Chinese HCC cohorts did not reveal any concurrence between the two mutations [34,46,53]. Another study that mined HCC data from TCGA and American HCC samples indicated a very low prevalence of *TERTp* mutations in NAFLD-HCC patients [111]. Clinical studies that found a significant association between *TERTp* and *CTNNB1* mutations tended to have a larger sample size than studies that did not identify a significant correlation (Table 4). Other potential reasons why the strength of the *TERTp-CTNNB1* association has varied between studies include racial or ethnic differences among the patients, different etiologies, and/or diverse disease course in HBV-endemic and non-endemic areas.

## 10. TERT and β-Catenin in Vertebrate HCC Models

The strong correlation between *TERTp* and *CTNNB1* mutations reported in large clinical studies of HCC have prompted a few groups to examine the *TERT-CTNNB1* cooperation using animal liver tumor models. Forced β-catenin overexpression in human fetal hepatocytes immortalized with hTERT (FH-hTERT) led to a malignant phenotype of loss of contact inhibition, increased anchorage-independent growth, and improved ability to form tumors in athymic nude mice [112]. These FH-hTERT pbcatS33Y cells have persistent clonal chromosomal translocations, which are not seen in FH-hTERT cells without β-catenin overexpression [112]. This finding indicates that the coexpression of TERT and β-catenin leads to tolerance for chromosomal structural instability, which may be important for the transformed phenotype. Additionally, using RNAi to knockdown *TERT* and *TERC*, this study showed that telomerase activity is not required for the short-term expansion of transformed cells [112].

Molina-Sánchez et al. showed cooperation between *TERT* and β-catenin in HCC with an elegant in vivo screen [113]. They used hydrodynamic tail-vein injections to deliver 23 different combinations of genetic alterations into mouse livers and examined the tumor formation six months later. Three out of nine mice overexpressing *Tert* and *CTNNB1* developed HCC, compared to zero of six mice overexpressing *CTNNB1* alone [113]. However, in a follow-up experiment (*n* = 9), no *Tert; CTNNB1* mice developed liver tumors [113]. The authors suggested that cooperation between *Tert* and β-catenin may be insufficient for HCC, and additional spontaneous genetic alterations may be required [113]. Future experiments in vertebrate models, including those combining both gain- and loss-of-function alterations in *TERT* and *CTNNB1*, will be critical for confirming and extending the results of Molina-Sánchez et al. Catalytically inactive TERT (TERT^ci^) constructs can be particularly useful in determining whether interactions between TERT and β-catenin require the telomerase activity of TERT or act through non-canonical mechanisms.

## 11. Conclusions and Future Directions

The key points from the above studies are: (1) *TERTp* and *CTNNB1* mutations are concordant in patient HCC; (2) in animal models, overexpression of *TERT* or *CTNNB1* promotes HCC, although the effect is modest in most models and often requires another oncogene or insult; (3) in cultured stem cells, *TERT* and *CTNNB1* cooperate via direct or indirect interactions during the transcription of target genes; and (4) both *CTNNB1* and, to a lesser extent, *TERT*, have key roles in cellular homeostasis, making the potent inhibition of endogenous genes unrealistic from a therapeutic perspective. 

Using cultured human liver cancer cells and vertebrate HCC models will be critical for confirming that the mechanism of TERT/β-catenin interaction identified in stem cells applies to HCC and establishing that non-canonical effects of TERT drive its interactions with β-catenin and the resulting HCC-promoting effects. Delineating exactly how TERT and β-catenin interplay would suggest promising treatment strategies for *TERTp* and *CTNNB1*-mutated HCC, including: (1) targeting non-canonical effects of *TERT*, critical for its tumor-promoting interactions with β-catenin; (2) targeting the interaction between TERT and β-catenin; and (3) combining relatively low doses of Wnt/β-catenin pathway and TERT inhibitors.

With respect to the latter approach, several drugs targeting either the WNT/β-catenin pathway or TERT are in early clinical trials or preclinical testing. The porcupine inhibitor CGX1321 and the Frizzled8 decoy receptor OMP-54F28 are currently in Phase I clinical trials [114,115]. The antibiotic and LRP5/6 inhibitor salinomycin and the Tankyrase inhibitor NVP-TNKS656 are in the preclinical stage for HCC [114,115]. All four of these drugs target WNT/β-catenin signaling upstream of β-catenin and, thus, may be ineffective in the setting of activating mutations in *CTNNB1*.

One option for inhibiting WNT/β-catenin signaling further downstream is to block β-catenin’s interactions with its transcriptional partners to prevent the expression of target genes. To that effect, molecules targeting β-catenin binding to CREB-binding protein (CBP) (PRI-724 (Phase 1 and 2)) or to TCF/LEF elements (LF3, iCRT3/5, ZINC02092166, and NLS-StAx-h; all in preclinical stages) are being tested [114,115]. CBP-inhibitors, such as ICG001 and Isoquercitrin, are also under preclinical evaluation [114].

Current strategies for the therapeutic targeting of TERT include: (1) small molecule inhibitors against TERT, like BIBR1532 [116]; (2) gene therapies against TERT, like telomelysin [117]; (3) gene silencing via microRNAs against TERT [117]; (4) G-quadruplex stabilizers that interfere with telomere structure and may inhibit telomerase activity, reviewed in detail by Alessandrini et al. [118]; (5) inhibitors of telomere- and telomerase-associated proteins, such as HSP90 [119]; (6) immunotherapies utilizing TERT as a tumor antigen [120]; and (7) oligonucleotides that mimic telomere overhangs (T-oligos) or inhibit telomerase (GRN163L) [117]. As with some of the WNT/β-catenin inhibitors, GRN163L shows promising anticancer activity, but its use as a single chemotherapeutic agent is limited by toxicity [117]. Combining a WNT/β-catenin inhibitor with a TERT inhibitor offers the potential to decrease serious side effects by using a lower dose of each drug.

The association of *CTNNB1* and *TERTp* mutations in HCC presents attractive diagnostic and prognostic possibilities. Risk stratification could be done based on the clues we have regarding the temporal play of gatekeeper and driver oncogenes. *TERTp* mutations are the first changes associated with neoplasia in cirrhotic livers. Thus, they could potentially be used to predict HCC risk in cirrhotic patients, just as β-catenin activation is currently used to predict HCC risk in patients with HCA. Further studies examining the prevalence of *TERTp* mutations in cirrhotic patients without HCC would be required to determine the feasibility of this approach.

HCC poses a therapeutic challenge due to the late diagnosis and paucity of effective treatments for advanced disease. The molecular heterogeneity in HCC further exacerbates this challenge, highlighting the need for increased identification and understanding of biomarkers and their prognostic and therapeutic value. Current HCC treatments are not tailored to patients based on the molecular features of their tumors, as no genetic perturbations have been definitively shown to influence therapeutic response in patients. As the armamentarium of approved HCC therapeutics expands, it will be critical to correlate molecular subtypes, including those with *TERTp* and *CTNNB1* mutations, with response to specific treatments to guide future patient treatment regimens.

## Figures and Tables

**Table 4:** Concordance between *TERTp* and *CTNNB1* mutations in HCC: clues from clinical studies.

Region	Etiology (N)	*TERTp* % Incidence (N)	*CTNNB1* % Incidence (N)	Association	Ref.
France	22% HBV+, 26% HCV+ (305)	59% (179)	33% (101)	Significant, *p* < 0.0001	[5]
France	20.7% HBV+, 26.5% HCV+ (801)	58.1% (441)	30.7% (229)	Significant, *p* = 0.0000001	[103]
Southern Italy	7.9% HBV+, 86.6% HCV+ (127)	50.4% (64)	26% (33)	Not significant, *p* = 0.4192	[43]
USA and Japan	25.6% HBV+, 42.6% HCV+ (469)	54.1% (254)	31.1% (146)	Significant, *p* < 0.0001	[7]
Japan	NAFLD (11)	82% (9)	45% (5)	Not significant, *p* = 0.4545	[54]
Taiwan	63% HBV+, 40.2% HCV+ (195)	29.2% (57)	16.5% (31/188)	Not significant, *p* = 0.2055	[34]
Korea	74.3% HBV+, 5.7% HCV+; (105)	39% (41)	14.6% (15)	Not significant, *p* = 0.568	[46]
China	83% HBV+ (190)	30% (57)	24.3% (17/70)	Not significant, *p* = 0.535	[53]

## Data Availability

Not applicable.
